# Comparing unconscious processing during continuous flash suppression and meta-contrast masking just under the limen of consciousness

**DOI:** 10.3389/fpsyg.2014.00969

**Published:** 2014-09-11

**Authors:** Ziv Peremen, Dominique Lamy

**Affiliations:** ^1^The School of Psychology Sciences, Tel Aviv University, Tel AvivIsrael; ^2^The Sagol School of Neuroscience, Tel Aviv University, Tel AvivIsrael

**Keywords:** conscious perception, unconscious perception, subliminal processing, meta-contrast masking, continuous flash suppression, response priming, awareness, consciousness

## Abstract

Stimuli can be rendered invisible using a variety of methods and the method selected to demonstrate unconscious processing in a given study often appears to be arbitrary. Here, we compared unconscious processing under continuous flash suppression (CFS) and meta-contrast masking, using similar stimuli, tasks and measures. Participants were presented with a prime arrow followed by a target arrow. They made a speeded response to the target arrow direction and then reported on the prime’s visibility. Perception of the prime was made liminal using either meta-contrast masking (Experiment 1) or CFS (Experiments 2 and 3). Conscious perception of the prime was assessed using a sensitive visibility scale ranging from 0 to 3 and unconscious processing was measured as the priming effect on target discrimination performance of prime-target direction congruency when prime visibility was null. Crucially, in order to ensure that the critical stimuli were equally distant from the limen of consciousness, we sought stimulus and temporal parameters for which the proportion of 0-visibility trials was comparable for the two methods. We found that the method used to prevent conscious perception matters: unconscious processing was substantial with meta-contrast masking but absent with CFS. These findings suggest that CFS allows very little perceptual processing, if at all, and that previous reports of high-level and complex unconscious processing during CFS may result from partial awareness.

## INTRODUCTION

Visual consciousness has been the focus of intense research in the last two decades (Marcel, 1983; Erdelyi, 1986, 2004; Greenwald et al., 1996; Vorberg et al., 2003; Ramsøy and Overgaard, 2004; Lau and Passingham, 2006; Schmidt and Vorberg, 2006; Lamy et al., 2009; Sandberg et al., 2010). The search for the limits of unconscious processing lies at the heart of this research: which processes can unfold in the absence of conscious perception and conversely, for which processes is consciousness essential? In other words, what is the function of consciousness? The most widely used empirical strategy used to address this question is to probe the influence on behavior of stimuli that are barred from conscious access, so as to assess what processes can be performed outside perceptual awareness.

A rather large arsenal of paradigms stand at disposal to prevent a visual stimulus from entering consciousness: pattern masking (e.g., Breitmeyer and Ganz, 1976), meta-contrast masking (Breitmeyer, 1978), object-substitution masking (Di Lollo et al., 2000), continuous flash suppression (CFS; Tsuchiya and Koch, 2005), the attentional blink (Raymond et al., 1992), inattentional blindness (Mack and Rock, 1998), and more (see Kim and Blake, 2005 for a review). The choice of the paradigm used to demonstrate unconscious processing often appears to be arbitrary, despite the fact that the different paradigms are known to affect perceptual processing in qualitatively different ways (e.g., Enns, 2004; Almeida et al., 2010; Kanai et al., 2010; Faivre et al., 2012; Fogelson et al., 2014). On the one hand, one could claim that the method used to prevent conscious perception should not matter as long as unconscious perception is demonstrated. On the other hand, however, it is important to minimize failures to identify processes that can be performed without consciousness. To do that, targeting the procedures that obliterate conscious processing while most minimally impairing unconscious processing would seem to be the most judicious strategy. To illustrate this point bluntly, blindfolding observers to prevent conscious perception would be a bad choice because it would also thoroughly eliminate unconscious processing.

In the present paper, we focused on a paradigm that has become increasingly popular in consciousness research: CFS (henceforth, CFS, Tsuchiya and Koch, 2005), and investigated the extent to which it disrupts unconscious processing. With this method, arrays of randomly generated shapes of different colors (Mondrians) presented successively at ∼10 Hz to one eye can reliably suppress the conscious awareness of an image presented to the other eye. One of the main reasons for the enthusiasm surrounding CFS is that, unlike backward masking, which is effective only when the target is presented very briefly (typically for less that 100 ms), CFS-induced suppression can last very long, on the order of seconds (Shimaoka and Kaneko, 2011; Stein et al., 2011). Based on the premise that high-level computations may require relatively long processing times, CFS should be a particularly well-suited paradigm in order to measure high-level unconscious processing.

Consistent with this conjecture, several studies relying on breaking suppression during CFS have revealed that we are capable of performing complex, high-level cognitive operations without conscious perception (e.g., Jiang et al., 2006; Costello et al., 2009; Mudrik et al., 2011; Sklar et al., 2012; Lupyan and Ward, 2013). In a nutshell, the rationale underlying the use of breaking suppression is that if a stimulus is found to overcome (or break) suppression earlier than another stimulus, then one can conclude that the property on which the two stimuli differ was processed unconsciously. However, there has been no convincing evidence that differences in breaking suppression times reflect genuine unconscious processing rather than processing under partial consciousness (e.g., Stein et al., 2011; see Gayet et al., 2014).

Other CFS studies that either used a traditional dissociation procedure or examined the neural consequences of this method have generated conflicting findings as to whether CFS interferes with low-level or with high-level cognitive processing (see Sterzer et al., 2014 for a review). For instance, some authors showed that subliminal stimuli suppressed using CFS elicit semantic processing (e.g., Almeida et al., 2008, 2010; Bahrami et al., 2010), while others showed that CFS-suppressed stimuli undergo only low-level perceptual processing (e.g., Faivre et al., 2012). Likewise, while several functional MRI studies showed robust suppression of activity in higher visual areas during CFS (e.g., Fang and He, 2005; Jiang et al., 2006; Hesselmann and Malach, 2011) but not in the primary visual cortex (Watanabe et al., 2011), Yuval-Greenberg and Heeger (2013) recently showed that CFS does in fact modulate fMRI responses in the primary visual cortex (see also Kang et al., 2011 for consistent findings using ERP methodology).

Most crucially, however, the very few studies that directly compared the extent of unconscious processing when stimuli are rendered invisible using CFS vs. other methods, suggest that CFS has the lower hand: it actually elicits more restricted unconscious processing (Almeida et al., 2010; Faivre et al., 2012, 2014; Izatt et al., 2014). For instance, Faivre et al. (2012) showed that while emotional face primes biased subsequent preference judgments when suppressed from awareness by gaze-contingent crowding, they did not elicit such emotion-related processing when suppressed by backward masking or CFS. Instead, they only produced an effect akin to low-level perceptual adaptation: responses to a face target were slower following an identical suppressed prime face relative to a suppressed face conveying the same emotional expression but displayed by a different individual. In addition, Almeida et al. (2010) showed that backward-masked primes elicited category- and identity-specific priming both with tool and with animal stimuli, whereas CFS-suppressed primes were associated only with small category-specific priming, and only with tool stimuli.

The foregoing studies relied on an objective measure of conscious perception to ensure that the prime was subliminal (but Izatt et al., 2014 used also a subjective measure). Specifically, using an experimental strategy that has become standard in the study of unconscious processing (Dehaene et al., 1998; Ansorge et al., 2009; Hsieh et al., 2011; Van Opstal et al., 2011) they included experimental trials in which the influence of a subliminal prime on responses to a subsequent target was probed, and prime-awareness test trials in which chance performance at judging the critical property of the prime was demonstrated. Thus, for instance, Almeida et al. (2010) showed that a suppressed prime facilitated response to a categorically congruent target, yet performance at discriminating the category to which this prime belonged was at chance.

**FIGURE 1:**
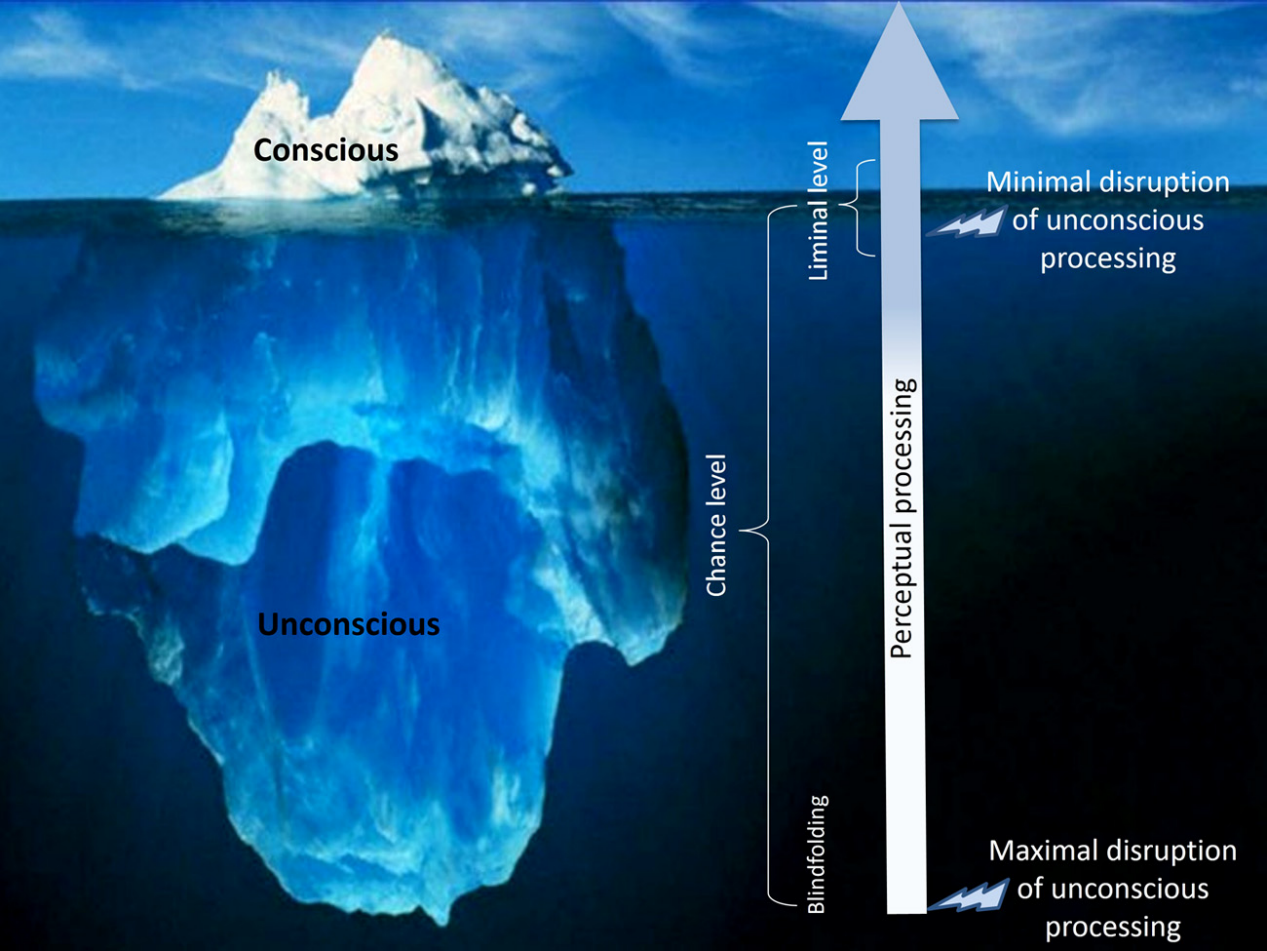
**Illustration of the ideas that (1) to maximize sensitivity for measuring unconscious processing, the critical stimulus must be as close as possible to the limen of consciousness and (2) chance performance at discriminating the critical stimulus is not informative with regard to this stimulus’ distance from the limen of consciousness**.

It is important to note that with objective measures of conscious perception, it is of tantamount importance to select stimuli that cannot be discriminated above chance: just a few visible trials can jeopardize the success of the whole experiment (e.g., Rouder et al., 2013). Thus, the safest strategy is to select deeply subliminal stimuli at the risk of “overshooting,” that is, of cutting into unconscious processing itself. However, the magnitude of such overshooting cannot be assessed because performance is undiscriminably at chance whether the critical stimulus is just under the limen or completely hidden from view (see **Figure 1**). As a consequence, finding that unconscious processing occurs using one method but not using another, may not necessarily reflect that these methods constitutively disrupt different stages of processing: instead, it might simply indicate that the stimulus parameters selected to ensure chance objective performance pushed perceptual processing further from the limen with one method relative to the other.

The objective of the present research was to assess the extent of unconscious processing using liminal stimuli instead of subliminal stimuli. We compared CFS with meta-contrast masking, a method that is thought to interfere with perceptual processing at a relatively late stage (e.g., Del Cul et al., 2007; Enns, 2004) and has been associated with robust priming (e.g., Vorberg et al., 2003; Kentridge et al., 2008; Peremen and Lamy, 2014). We assessed conscious perception of the prime using a sensitive subjective visibility scale akin to the Perceptual Awareness Scale (e.g., Ramsøy and Overgaard, 2004)^1^. One of the main advantages of using this measure in the present context is that it allows using *liminal* stimuli, that is, stimuli that are subjectively invisible on some proportion of the trials and perceived at various degrees of clarity on other trials. In this way, one can prevent conscious processing while minimally encroaching on unconscious processing. In addition to minimizing the distance of the critical stimulus perception from the limen of consciousness, visibility scales allow one to measure this distance – a feature that is particular useful when comparing different methods for preventing conscious perception: similar proportions of invisible trials should indicate similar distances from the limen.

In the present study, we compared the extent of unconscious processing of a prime arrow direction when this arrow was rendered invisible using meta-contrast masking (Experiment 1)^2^, or CFS (Experiments 2 and 3). In all three experiments, participants were presented with a liminal prime arrow followed by a clearly visible target arrow, the direction of which was either congruent or incongruent with the prime arrow direction. On each trial, participants first made a speeded forced-choice discrimination response to the direction of the target arrow and then rated the visibility of the prime on a scale ranging from 0 to 3. Unconscious processing of the prime arrow direction was measured as the performance difference between the congruent and incongruent conditions on trials in which participants reported their subjective visibility of the prime to be null.

## EXPERIMENT 1

### METHODS

#### Participants

Twenty two right-handed undergraduate students from Tel Aviv University (13 women), age 22–28 years (*M* = 24.9, SD = 1.9) were tested in one session for course credit. All subjects reported normal or corrected-to-normal vision.

#### Apparatus and stimuli

**FIGURE 2:**
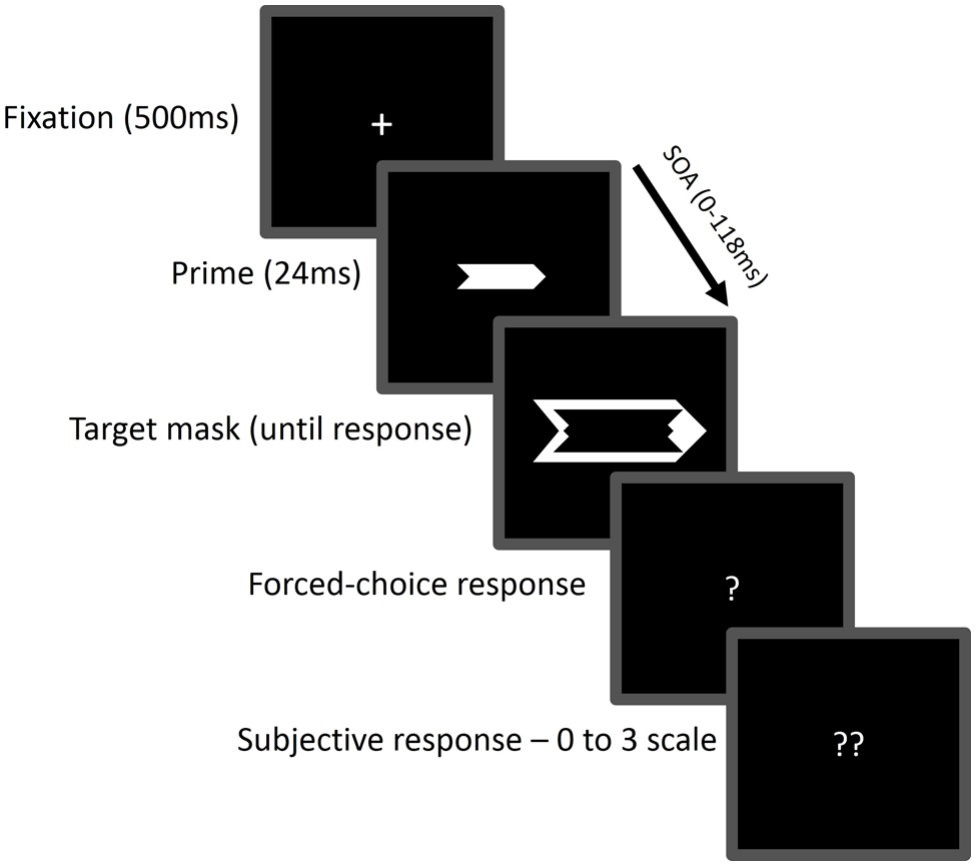
**Sequence of events in Experiment 1.** In this example, the directions of the prime and target arrows are congruent. Participants were required to make a speeded response to the target mask arrow direction (left or right) and then rate subjective visibility of the prime.

Sample displays are presented in **Figure 2**. The stimuli were presented on a 17-inch 85-Hz CRT monitor. The fixation display consisted of a plus sign (0.2^∘^× 0.2^∘^ of visual angle). The prime display consisted of a small arrow (1.6^∘^× 0.8^∘^) and the target-mask display consisted of a larger arrow, 2.1^∘^ in width and 1.1^∘^ in height. Both arrows were gray (RGB 127, 127, 119) against a black background (RGB 0, 0, 0), were centered at fixation and pointed either leftward or rightward. Thus, the prime arrow either pointed in the same direction as the target arrow (congruent trials) or in the opposite direction (incongruent trials).

#### Procedure and design

Each trial began with a 500-ms presentation of the fixation display. The prime display then appeared for 24 ms, followed after a variable SOA (0, 24, 47, 71, 94, or 118 ms) by the target-mask display. Then, a blank screen appeared until subjects provided the first response or after 2,000 ms had elapsed, followed by a question mark in the middle of the screen, which prompted the subjects to provide the second response. A new trial began immediately after second response.

On each trial, subjects provided two responses: they first made a speeded response to the target-mask arrow direction by pressing designated keys as fast as possible on the numerical keypad with their right hands (“1” when the arrow pointed to the left and “3” when it pointed to the right). Then, they provided a subjective report of the prime visibility using a scale ranging from 0 (“I saw nothing at all”) to 3 (“I saw the arrow clearly”) by pressing designated keys (“z,” “x,” “c,” and “v” which were covered with stickers labeled 0, 1, 2, and 3, respectively) on the keyboard with their left hands. Five percent of the trials were catch trials: the target was presented alone, without a prime. The purpose of introducing catch trials was to anchor 0-visibility judgments to situations in which no prime appeared. On 10% of the trials (*no-go* trials) the target arrowheads were truncated and observers had to press the space-bar instead of providing the two responses pertaining to the prime^3^. Each subject completed 500 trials divided into ten blocks and following two practice blocks of 50 trials each. Before practice, the observers viewed the sequence of events at a very slow pace that enabled them to clearly distinguish between the prime and target.

All combinations of the prime and target arrow directions were equiprobable and randomly mixed. They were equally likely to be congruent or incongruent. Prime-to-target SOAs were equiprobable and randomly mixed.

### RESULTS

**FIGURE 3:**
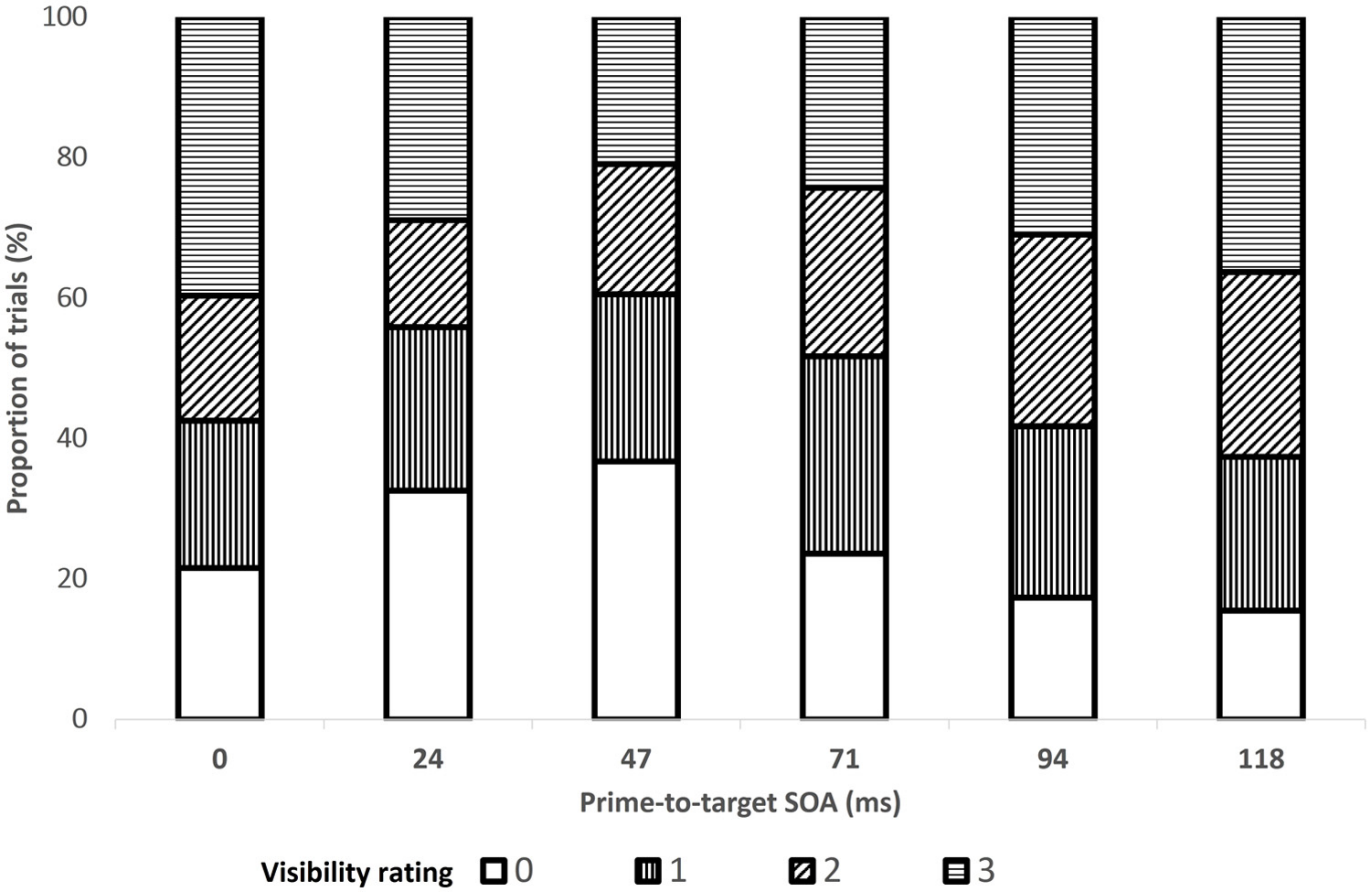
**Proportion of trials in each level of prime visibility (0–3) as a function of the SOA between the prime and target, in Experiment 1**.

The data from two participants were excluded from analysis: one because his mean RTs were slower than the group’s by more than 3 SDs and the other, because of a technical error. Prime-absent (or catch) trials as well as *no-go* trials were excluded from all analyses. In all RT analyses, trials in which responses to the target direction were inaccurate were excluded (2.3%) and so were trials in which the RT exceeded the mean of its cell (resulting from crossing the factors included in the relevant analysis) by more than 2.5 SDs (fewer than 1% of the trials). An ANOVA with SOA as a within-subject factor and mean visibility as the dependent measure revealed that mean visibility followed the U-shaped pattern characteristic of type-B meta-contrast masking (Kolers and Rosner, 1960) and was lowest at the 47-ms SOA (this trend did not reach significance after Huynh–Feldt correction, *F*(5,75) = 2.69, *p* < 0.09). The mean proportion of trials per visibility for each SOA is shown in **Figure 3**^4^.

#### Reaction times

**FIGURE 4:**
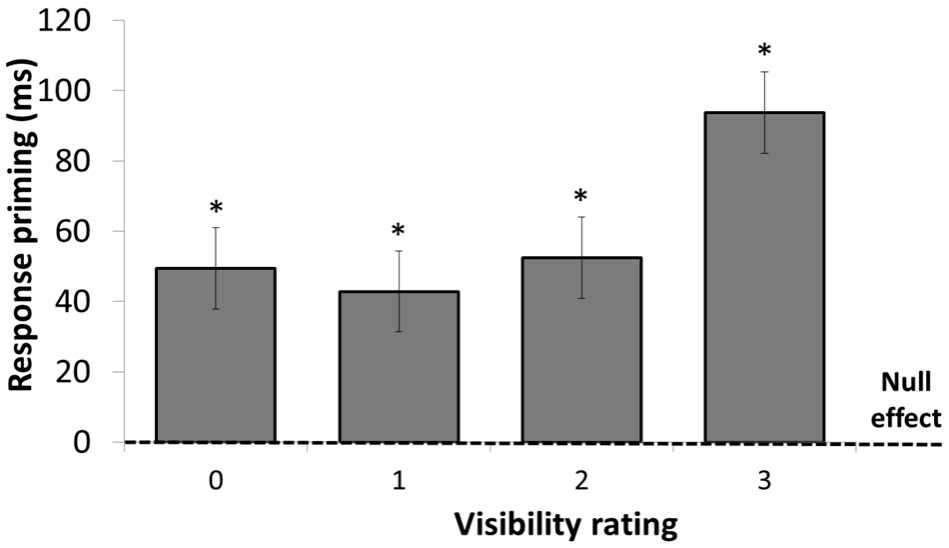
**Mean response-priming effect in milliseconds in Experiment 1, as a function of visibility rating.** Priming was significant for visibility 0–3. Error bars represent standard errors. *p < 0.002.

A linear mixed-effects model with visibility (0, 1, 2, or 3) and congruency (congruent vs. incongruent) as within-subject factors was performed on the mean RTs. Mean RT and accuracy data are presented in **Table 1** and the mean congruency effect at each visibility level is shown in **Figure 4**^5^. The main effect of congruency was significant, *F*(1,19) = 86.94, *p* < 0.0001, with faster RTs when the prime and target arrows were congruent than when they were incongruent. The main effect of visibility was also significant, *F*(3,53) = 32.16, *p* < 0.0001, with longer RTs as visibility increased. The interaction between the two factors was significant, *F*(3,53) = 3.7, *p* < 0.02. Further analyses revealed that the congruency effect was larger for visibility three than for all other levels, all *p*s > 0.03 and that the congruency effects for visibility levels 0, 1, and 2 did not differ from each other, all *F*s < 1. Crucially, the congruency effect was significant when visibility was null, 49 ms *F*(1,53) = 14.82, *p* < 0.001.

**Table 1:** Mean reaction times and accuracy on congruent and on incongruent trials in Experiment 1 as a function of visibility rating.

	Reaction times (ms)		Accuracy (%)	
Visibility	Congruent	Incongruent	Congruent	Incongruent
0	606.2	655.6	98.7	97.8
1	640.9	683.8	99.0	98.2
2	693.7	746.2	98.0	98.2
3	653.5	747.3	97.0	95.9

#### Accuracy

Similar analyses were conducted on the accuracy data pertaining to the responses to the target arrow. They showed similar trends, thus ensuring that speed-accuracy trade-off was not a concern. The main effect of visibility was significant, *F*(3,53) = 6.81, *p* < 0.001, the main effect of congruency approached significance *F*(1,19) = 3.75, *p* < 0.07 and the interaction between the two factors was not significant, *F* < 1.

## EXPERIMENT 2

### METHODS

#### Participants

Fifteen undergraduate students from Tel Aviv University (fourteen right-handed, 11 women,), age 20–27 years (*M* = 23, SD = 1.65) were tested in one session for course credit. All subjects reported normal or corrected-to-normal vision.

#### Apparatus, stimuli, procedure, and design

The apparatus was the same as in Experiment 1 except for the following changes. All stimuli were presented on a LCD monitor (SyncMaster) with 1920 × 1080 resolution, 120 Hz refresh rate controlled by a Power Samsung 3D PC. In order to create stereoscopic perception the stimuli were viewed through SSG-M3150GB 3D Active Glasses (battery powered), which let one image through the left eye while blocking stimulation to the right eye and another image to the right eye while blocking stimulation to the left eye, with a 60-Hz alternation rhythm that is beyond the perceptual threshold. The target display was presented together with the Mondrian suppressors to one eye, whereas the prime display was displayed to the other (“suppressed”) eye.

**FIGURE 5:**
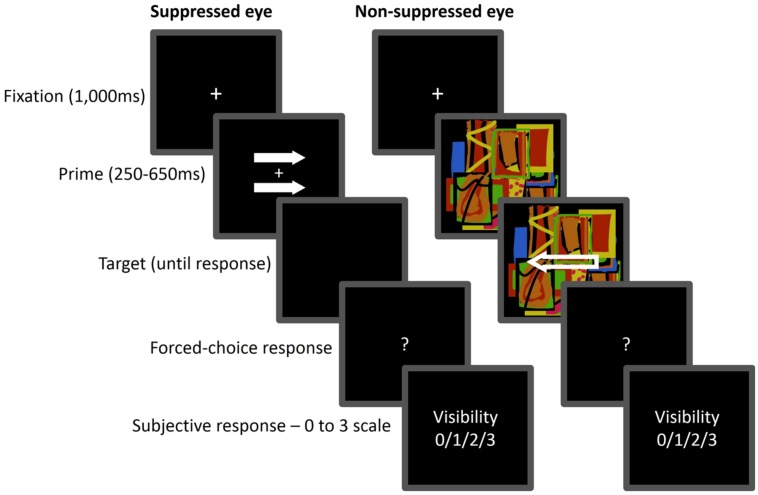
**Sequence of events in Experiment 2.** The prime arrows were gradually introduced and presented to one eye (suppressed eye), while the target was superimposed on a dynamic Mondrian and presented to the other eye (non-suppressed eye). Here, the directions of the prime and target arrows are incongruent. Participants were required to make a speeded response to the target arrow direction (left or right) and then rate subjective visibility of the prime.

Sample displays are presented in **Figure 5**. The prime display consisted of two filled horizontal arrows (1.72^∘^ × 0.46^∘^ each) pointing in the same direction, either left or right, and presented 0.57^∘^ above and below the center of the screen. The two prime arrows were gray and appeared at variable contrast levels of 20, 60, or 100% of maximum contrast level (RGB 195, 195, 195). The target display consisted of a horizontal white outline arrow (1.72^∘^× 0.57^∘^) pointing either leftward or rightward. All arrows were presented against a black background. The suppressors were Mondrians, that is, randomly colored figures of partly overlapping rectangles of varying sizes and colors. A white rectangular frame (18.16^∘^ × 18.16^∘^) centered at fixation was presented to each eye throughout the trial.

Each trial began with a 1,000-ms presentation of the fixation display. The prime display was then faded in by ramping up its contrast from 0% to a contrast level of 20, 60, or 100% in 200 ms. It remained on the screen until the target was presented, following a variable SOA (250, 350, 450, 550, or 650 ms). The target display remained visible until response. The subsequent events as well as the response requirements were the same as in Experiment 1.

The two prime arrows and the target arrow were equally likely to point to the left or right, and were therefore equally likely to be congruent or incongruent. Conditions of prime-arrows direction, target-arrow direction, SOA and prime contrast were randomly mixed.

### RESULTS

In all RT analyses, trials in which responses to the target arrow direction were inaccurate were excluded (1.2%) and so were trials in which the RT exceeded the mean of its cell by more than 2.5 SDs (fewer than 1.6% of the trials).

**FIGURE 6:**
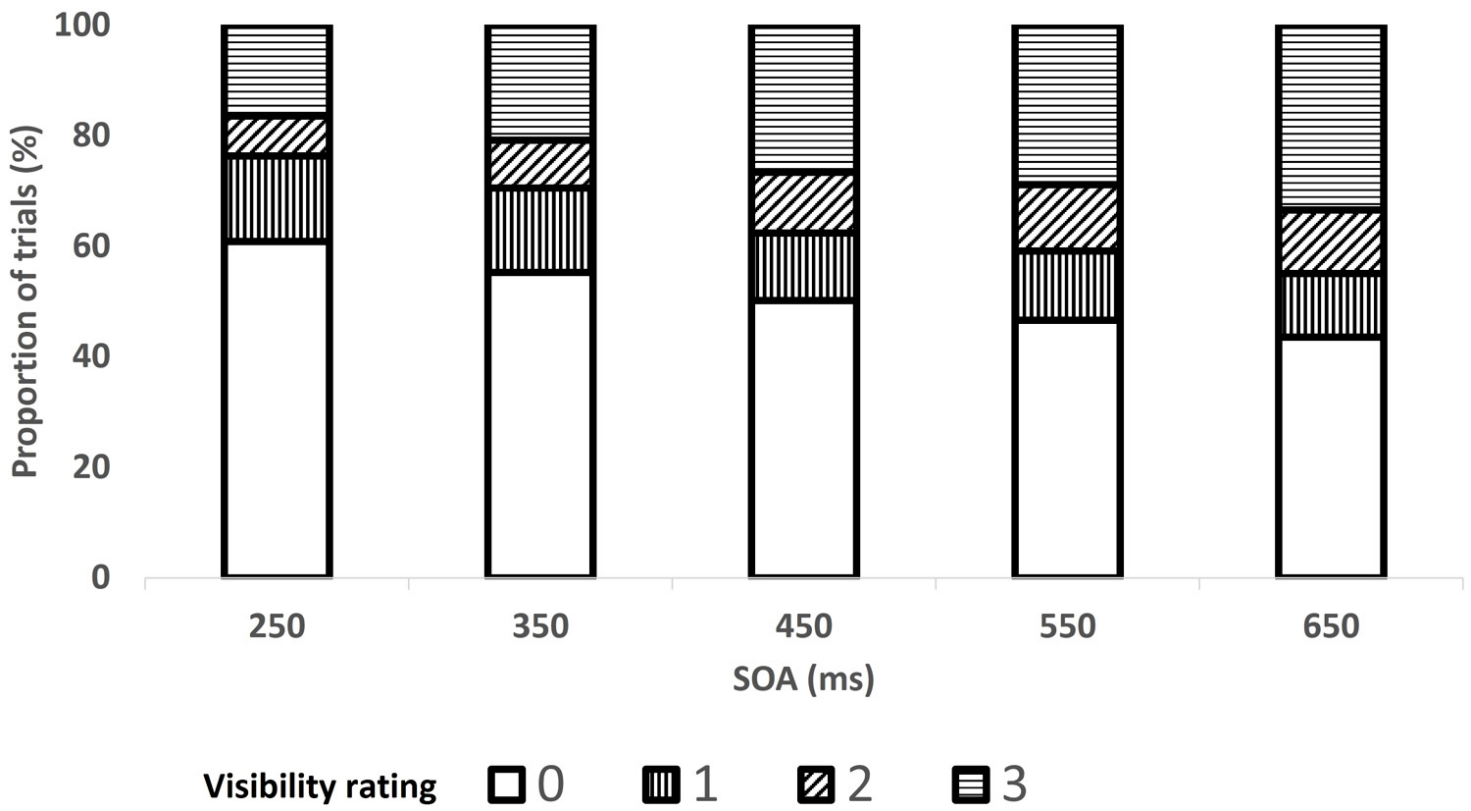
**Proportion of trials in each level of prime visibility (0–3) as a function of the SOA between the prime and target, in Experiment 2**.

**FIGURE 7:**
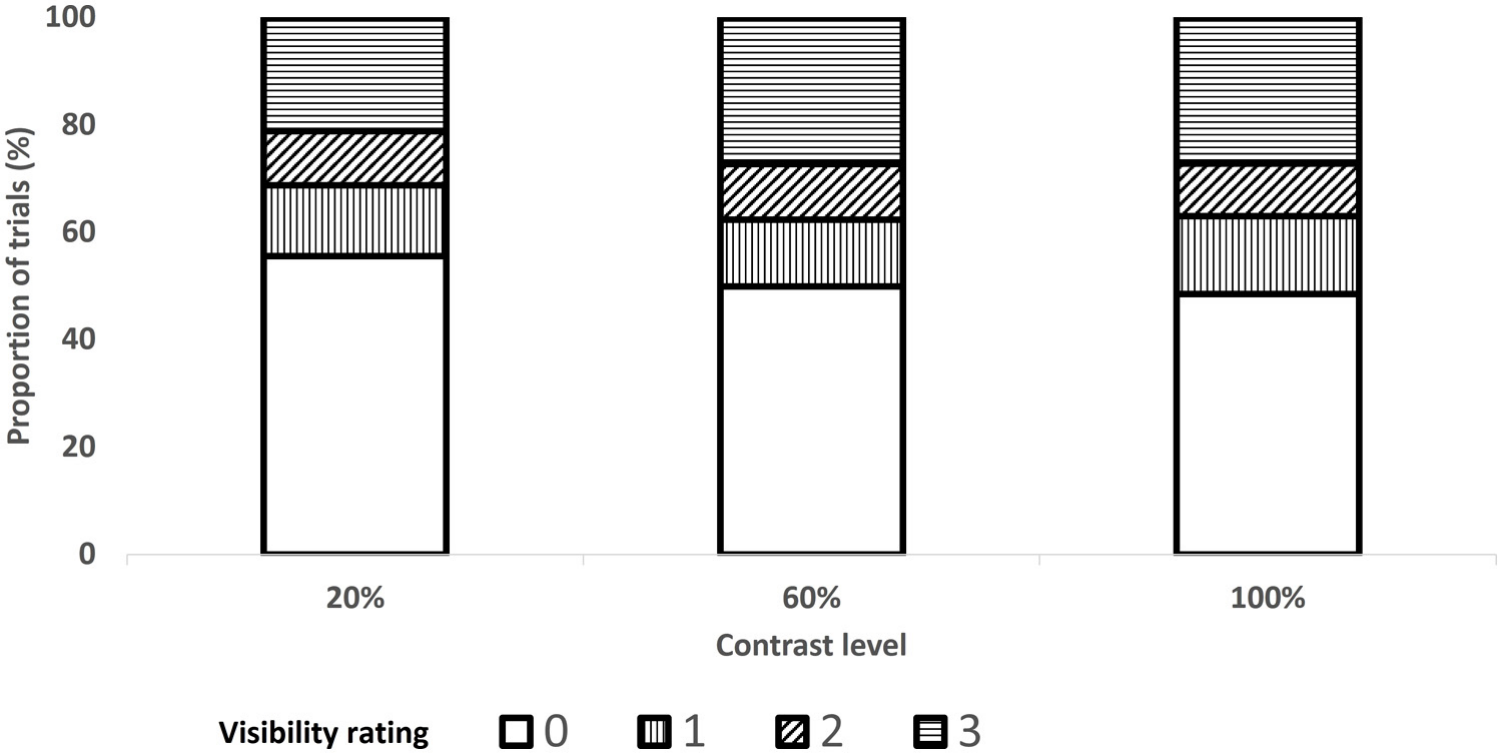
**Proportion of trials in each level of prime visibility (0–3) as a function of prime contrast level, in Experiment 2**.

An ANOVA with SOA and prime-contrast level as within-subject factors and mean visibility as the dependent variable revealed significant main effects, *F*(4,56) = 18.51, *p* < 0.0001 and *F*(2,28) = 17.16, *p* < 0.0001, respectively, with higher visibility as the SOA and prime-contrast increased. The significant interaction between these factors, *F*(8,112) = 4.87, *p* < 0.002 indicated that the effect of prime contrast became significant only for SOAs exceeding 350 ms. The mean proportions of trials per visibility is shown in **Figure 6** as a function of SOA and in **Figure 7** as a function of prime-contrast level.

#### Reaction times

**FIGURE 8:**
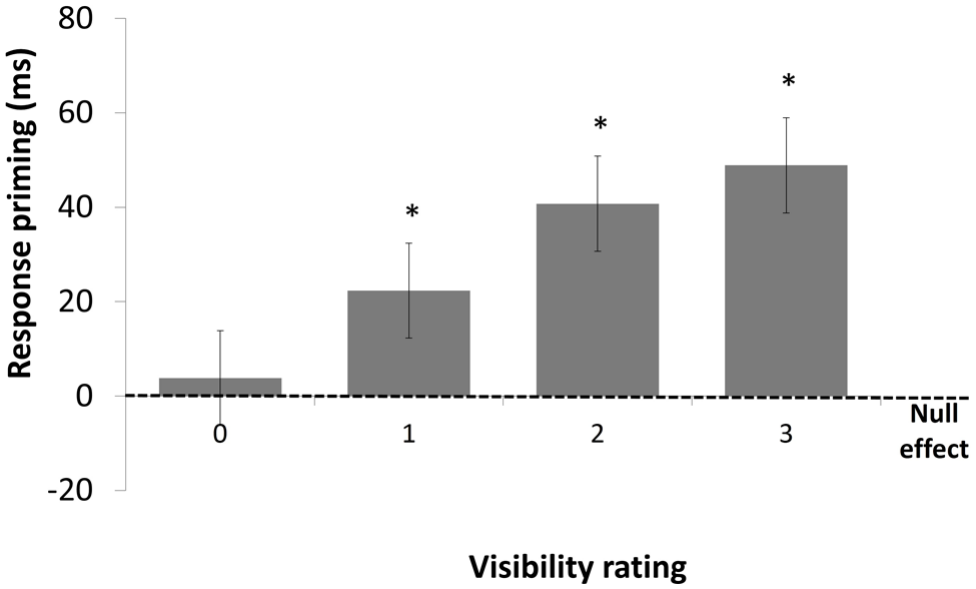
**Mean response-priming effect in milliseconds in Experiment 2, as a function of visibility rating.** Priming was significant for visibility 1, 2, and 3, but not when visibility was null. Error bars represent standard errors. *p < 0.03.

A linear mixed-effects model with visibility (0, 1, 2, or 3), congruency (congruent vs. incongruent), prime-contrast level (20, 60, 100%) and SOA (250, 350, 450, 550, or 650 ms) as within-subject factors was performed on the mean RTs of correct trials. The main effect of SOA was significant, *F*(4,56) = 22.95, *p* < 0.0001 with slower RTs as SOA increased and did not interact with congruency, *F* < 1. There was no significant effect of prime contrast F(2,28) = 1.99, *p* = 0.16, and no interaction involving this factors, all *F*s < 1. Mean RTs and accuracy data are presented in **Table 2** and the mean congruency effect at each visibility level is shown in **Figure 8**. The main effect of congruency was significant, *F*(1,14) = 41.39, *p* < 0.0001 with faster RTs when the directions of the prime and target arrows were congruent than when they were incongruent. The main effect of visibility was also significant, *F*(3,40) = 104.28, *p* < 0.0001, indicating that RTs were slower for 0- than for 1-, 2- and 3-visibility trials, all *p*s < 0.0002. There was a significant interaction between the two factors, *F*(3,38) = 8.04, *p* < 0.0003. Further analyses revealed that the congruency effect was significant for visibility levels 1, 2 and 3, *F*(1,38) = 5.06, *p* < 0.03, *F*(1,38) = 12.6, *p* < 0.001 and *F*(1,38) = 45.12, *p* < 0.001, respectively but crucially and unlike the pattern of results observed in Experiment 1, response priming was not significant when visibility was null, *F* < 1. As is clear from **Figure 8** response priming increased linearly with increasing levels of visibility.

**Table 2:** Mean reaction times and accuracy on congruent and on incongruent trials in Experiment 2 as a function of visibility rating.

	Reaction times (ms)		Accuracy (%)	
Visibility	Congruent	Incongruent	Congruent	Incongruent
0	685.5	689.3	99.5	99.0
1	757.1	779.5	99.0	97.7
2	750.4	791.1	99.5	96.4
3	697.7	746.5	99.2	97.9

#### Accuracy

Similar analyses conducted on the accuracy data showed similar trends. The main effect of visibility was significant, *F*(3,37) = 3.82, *p* < 0.02. No effect involving congruency approached significance, all *p*s > 0.2.

### DISCUSSION

The results of Experiments 1 and 2 revealed a markedly different pattern. When the prime was invisible, response priming was significant when invisibility was achieved using meta-contrast masking (Experiment 1), but was absent when invisibility was achieved using CFS (Experiment 2). These findings suggest that CFS interferes with processing more deeply than does meta-contrast masking. However, four alternative accounts must be considered.

First, although the stimuli used were largely similar in the two experiments, they differed in a few respects. For instance, the prime was one central arrow in Experiment 1 and two eccentric (albeit foveal) arrows in Experiment 2. Although stimulus-related differences should not matter as long as the primes are liminal to the same extent, one could claim that there might be qualitative differences in unconscious processing of central and eccentric stimuli.

Second, when the prime was clearly visible (visibility 3), response priming was larger in the meta-contrast than in the CFS experiment (93 vs. 49 ms, respectively). Thus, it may be the case that the meta-contrast masking paradigm yielded larger response priming overall in our experiment and was therefore more sensitive for detecting unconscious processing than was the CFS paradigm. In order to test this possibility, we analyzed the data from participants who showed the *largest* response priming effects for maximum visibility in the CFS experiment (i.e., with response priming exceeding the median effect of the group, 57 ms). The mean congruency effect for visibility-3 trials in large-response-priming participants was 78 ms, and was therefore similar to the mean congruency effect in Experiment 1 (93 ms). Yet, response priming when the prime was invisible remained null (4 ms, *F* < 1). Conversely, we analyzed the data from participants who showed the *smallest* response priming effects for maximum visibility in the meta-contrast masking experiment (i.e., response priming effect lower than the median effect of the group, 75 ms). The mean congruency effect for visibility-3 trials in small-response-priming participants was 54 ms, and was therefore similar to the mean congruency effect in Experiment 2 (49 ms). Yet, in line with our prediction, response priming was still highly significant when the prime was invisible [75 ms, *F*(1,23) = 21.86, *p* < 0.0001]. We can thus conclude that differences in the magnitudes of response priming on high-visibility trials does not account for the observed differences in unconscious processing between the two methods.

Third, as the distribution of trials across visibility levels varied as a function of SOA (**Figure 6**) and prime contrast (**Figure 7**), our finding of a large congruency effect when the prime was visible (ratings 1, 2, and 3) but not when it was invisible (rating 0) may reflect SOA- or prime-contrast- rather than visibility-related differences. In other words, the null response priming effect on 0-visibility trials may mainly emanate from short-SOA or low-prime-contrast trials, whereas the large response priming effect on visibility-3 trials may mainly emanate from long-SOA or high-prime-contrast trials (note that this problem does not apply to Experiment 1 because priming was found for visibility 0). The fact that the congruency effect interacted with neither SOA nor contrast level is inconsistent with such a claim, yet we nevertheless conducted additional analyses to examine this possibility. We focused only on the SOA (250 ms) and prime-contrast level (20%) for which visibility three ratings were least frequent. The congruency effect was still present for visibility-3 trials, *F*(1,32) = 3.58, *p* < 0.07 and *F*(1,32) = 4.93, *p* < 0.04, for the 250-ms SOA and 20%- prime contrast, respectively, and still absent for visibility-0 trials, both *F*s < 1.

The fourth alternative account rests on the observation that the proportion of 0-visibility trials was overall larger in the CFS than in the meta-contrast experiment. The lowest proportion of such trials was 44% (with the 650-ms SOA) in the former, whereas the highest proportion in the latter experiment was 37% (with the 47-ms SOA). As explained in the introduction, such a state of affairs indicates that the prime stimuli were further from the limen of consciousness in the CFS than in the meta-contrast experiment, which could explain why we failed to observe unconscious processing with CFS.

Although the additional analyses reported above provide a partial answer to the second and third issues, the objective of Experiment 3 was to address all four issues more directly.

## EXPERIMENT 3

In this experiment, we again used CFS to manipulate conscious perception of the prime but introduced three changes in order to test our conclusions from Experiment 2 against alternative accounts. First, the prime and target arrows were now identical to those used in Experiment 1, so as to preclude any account based on stimulus-based differences between the meta-contrast and CFS experiments. Second, to ensure that the unconscious and conscious conditions were physically identical, we used only one contrast level and one prime-target SOA. Third, we selected a high prime-contrast level and ramped it up faster than in Experiment 2 in order to bring the prime stimulus to a closer distance to the limen. Specifically, we aimed at obtaining a percentage of 0-visibility trials similar or smaller than for the SOA associated with the highest such percentage in the meta-contrast experiment (47 ms), in which a significant priming effect was observed.

Note that while we physically equated the prime and target stimuli in Experiments 3 and 1, we used different SOAs (from 0 to 118 ms in Experiment vs. 200 ms in Experiment 3). Obviously, stimulus conditions to prevent consciousness are going to be different in any two methods, in order for these methods to be distinguished: had we used exactly the same stimuli and SOAs in the CFS as in the meta-contrast experiments, prime stimuli would have suffered from both meta-contrast masking and CFS. Consistent with this observation, it is noteworthy than none of the previous studies which compared CFS and backward masking used identical stimuli or temporal parameters. For instance, Faivre et al. (2012) presented the critical primes for 2,500 ms with CFS and for 50 ms with backward masking. Likewise, Almeida et al. (2010) presented their primes twice for 100 ms in the CFS condition and once for 35 ms in the backward masking condition. However, in Experiment 3, in order to minimize the potential consequences of using long prime durations with CFS, we selected a relatively short SOA, namely, 200 ms, which ensured that no meta-contrast masking could occur (e.g., Enns, 2004). If the findings of Experiment 2 resulted from genuine differences between CFS and meta-contrast masking rather than from any of the four alternative accounts we suggested then we should expect to replicate these findings in the present experiment.

### METHODS

#### Participants

Thirteen undergraduate students from Tel Aviv University (12 right-handed, eight women), age 20–28 years (*M* = 24.0, SD = 2.4) were tested in one session for course credit or for a 30-NIS pay (∼8USD). All subjects reported normal or corrected-to-normal vision.

#### Apparatus, stimuli, procedure, and design

**FIGURE 9:**
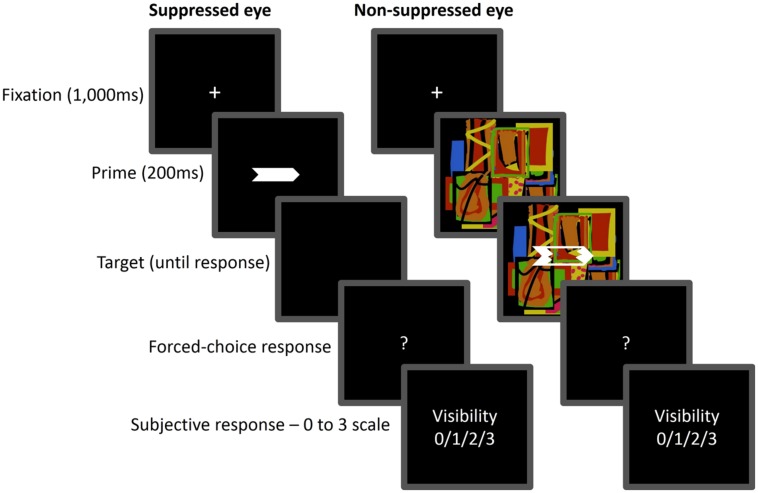
**Sequence of events in Experiment 3.** Prime-to-target SOA was fixed at 200 ms and prime contrast level also fixed.

The apparatus, stimuli, procedure and design were the same as in Experiment 2 except for the following changes. First, the prime and mask arrows were exactly the same as in Experiment 1. Sample displays are presented in **Figure 9**. Second, prime contrast was not manipulated: on each trial, the prime display was faded in by ramping up its contrast to 100% of maximum contrast level in 50 ms. Finally, the target display followed the prime after a fixed SOA of 200 ms.

### RESULTS AND DISCUSSION

**FIGURE 10:**
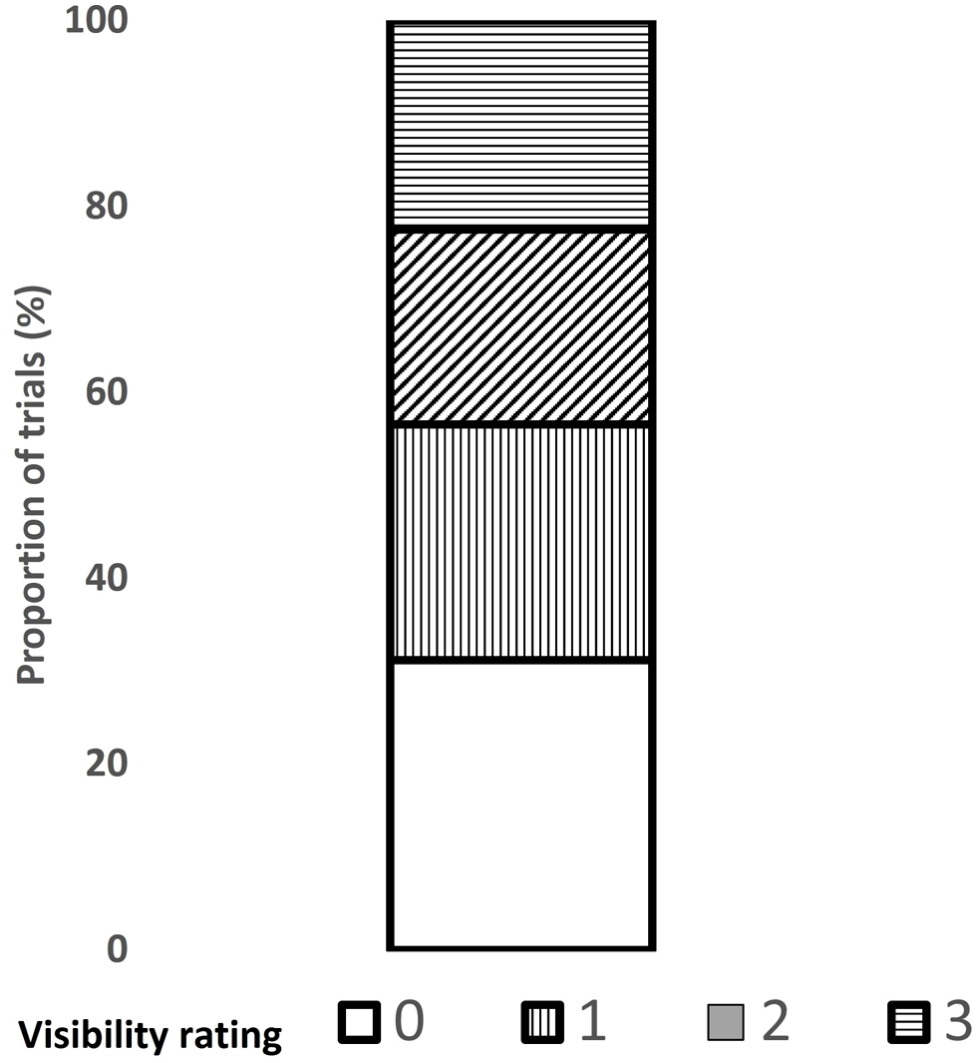
**Proportion of trials for each level of prime visibility (0–3) in Experiment 3**.

The data from one participant was excluded from analysis because upon debriefing, he reported using the strategy of foveating the periphery of the display, which helped him perceive the prime more easily. Prime-absent (or catch) trials as well as *no-go* trials were excluded from all analyses. In all RT analyses, trials in which responses to the target arrow direction were inaccurate were excluded (1.9%) and so were trials in which the RT exceeded the mean of its cell by more than 2.5 SDs (fewer than 2.4% of the trials). The mean proportions of trials per visibility are shown in **Figure 10**.

#### Reaction times

**FIGURE 11:**
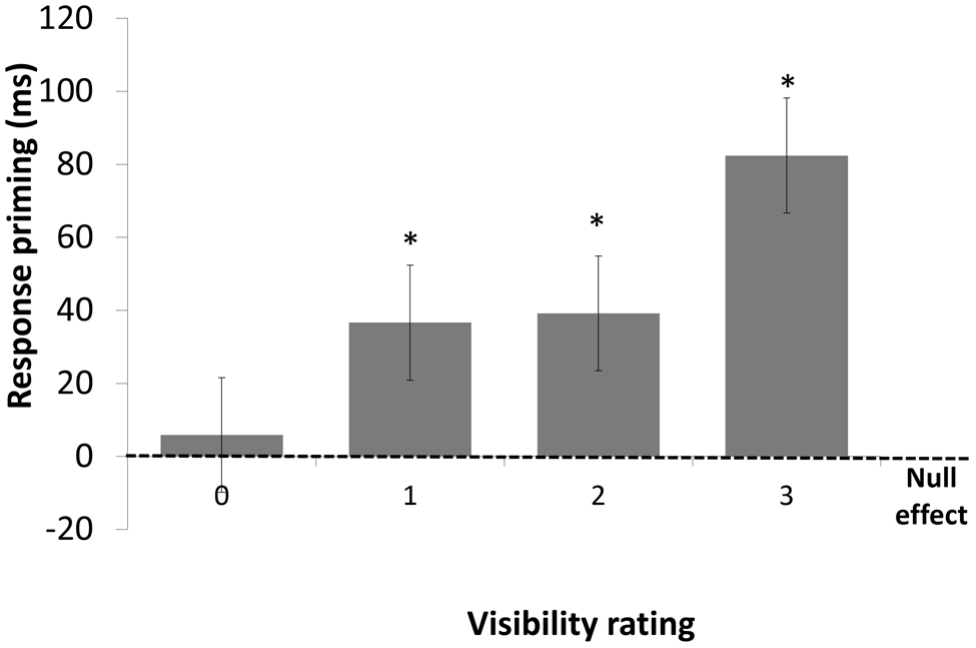
**Mean response-priming effect in milliseconds in Experiment 3, as a function of visibility rating.** Priming was significant for visibility 1, 2, and 3, but not when visibility was null. Error bars represent standard errors. *p < 0.001.

A linear mixed-effects model with visibility (0, 1, 2, or 3) and prime-target congruency (congruent vs. incongruent) as within-subject factors was performed on the mean RTs of correct trials. Mean RTs are presented in **Table 3** and the mean congruency effect at each visibility level is shown in **Figure 11**. The main effect of congruency was significant, *F*(1,11) = 159.26, *p* < 0.0001, with faster RTs when the directions of the prime and target arrows were congruent than when they were incongruent. The main effect of visibility was also significant, *F*(3,33) = 12.83, *p* < 0.0001, indicating that RTs became slower as mean visibility ratings increased. There was a significant interaction between the two factors, *F*(3,32) = 24.74, *p* < 0.0001. Closely replicating the findings of Experiment 2, the congruency effect increased as visibility increased and was significant for visibility levels 1, 2, and 3, *F*(1,32) = 33.0, *p* < 0.001, *F*(1,32) = 31.19, *p* < 0.001, and *F*(1,32) = 147.74, *p* < 0.001, respectively. Crucially, however, it was again non significant when visibility was null, *F* = 1.03, *p* = 0.32.

**Table 3:** Mean reaction times and accuracy on congruent and on incongruent trials in Experiment 3 as a function of visibility rating.

	Reaction times (ms)		Accuracy (%)	
Visibility	Congruent	Incongruent	Congruent	Incongruent
0	669.3	675.2	99.1	98.8
1	653.2	689.8	99.3	98.9
2	672.8	712.0	99.2	99.0
3	654.2	736.6	98.8	98.2

#### Accuracy

Similar analyses were conducted on the accuracy data. The congruency effect was not significant, *F*(1,11) = 2.25, *p* = 0.16 and neither were all other effects, all *F*s < 1.

The results replicated the findings of Experiment 2, yet they can be accounted for by none of alternative interpretations raised with respect to Experiment 2. In particular, the magnitude of response priming on maximum-visibility trials was similar to the one observed with meta-contrast masking (Experiment 1). In addition, the proportion of null-visibility trials was smaller in this experiment than in Experiment 2 and was now similar to the proportion observed in the meta-contrast experiment. In fact, this proportion was smaller here (31.2%) than with the 24 and 47-ms SOAs (32.6 and 36.7%, respectively), for which significant response priming was observed in Experiment 1. Thus, the stimuli were unlikely to be further from the limen of consciousness in the CFS relative to the meta-contrast experiment.

## GENERAL DISCUSSION

In this study, we compared unconscious processing under meta-contrast masking and CFS. Conscious perception was assessed using a sensitive visibility scale ranging from 0 to 3 and unconscious processing was measured as a significant effect of the congruency between the directions of a prime and target arrows when participants reported not seeing the prime at all (i.e., when its visibility was rated to be 0). The central finding is that unconscious processing was substantial with meta-contrast masking but absent with CFS.

Although previous studies have also compared different suppression methods and shown that CFS allows only little unconscious processing, it is important to report conceptual replications of these findings on the backdrop of the increasing popularity of CFS as a tool to study unconscious processing. We extend previous findings by comparing CFS to meta-contrast masking rather than pattern backward masking or gaze-contingent crowding, and by probing unconscious response priming that relies on simple shape perception, rather than semantic category discrimination or emotional processing (Almeida et al., 2010 and Faivre et al., 2012, respectively). In addition, our comparison involved exactly the same prime and target stimuli unlike Almeida et al. (2010) who added 70% of noise to the prime stimuli in the masking but not in the CFS experiment and Faivre et al. (2012) study who cropped peripheral facial attributes (e.g., hair, ears) in the masking but not in the CFS experiment. Finally and most importantly, we used a novel methodology to ensure that the critical stimuli were at a comparable distance from the limen of consciousness during CFS and meta-contrast masking.

### COMPARISON WITH PREVIOUS CFS STUDIES

The finding that CFS disrupts relatively low-level perceptual processes calls for a reappraisal of previous demonstrations that highly complex processing can be performed when conscious perception of the critical stimuli is prevented using CFS. Therefore, our study accredits the notion that unconscious processing demonstrated by measuring the time of breaking of CFS suppression (e.g., Jiang et al., 2006; Costello et al., 2009; Mudrik et al., 2011; Sklar et al., 2012; Lupyan and Ward, 2013) resulted from partial awareness of the suppressed stimuli (e.g., Stein et al., 2011; Gayet et al., 2014). However, our results appear to be at odds with previous reports of unconscious priming during CFS.

Bahrami et al. (2010) examined whether invisible numerical stimuli could prime a visible numerical target. They measured subjective awareness on a scale ranging from 0 to 2 on each trial and reported a significant effect of the numerical distance between the prime and target on 0-prime-visibility trials. However, unconscious numerical processing was very tenuous. The priming effect, measured as the RT-difference between prime-present and prime-absent trials, was found to depend on the identity of the prime only for one specific prime-target distance: RTs were faster for same than for different prime-target trials only for the prime-target distance of -2 (and not for distances of -1, 1, and 2).

Almeida et al. (2010) reported a small (<15 ms), yet significant category-specific priming effect for tool vs. animal stimuli with invisible primes. CFS-suppressed stimuli were held to be invisible based on an awareness pre-test which determined the individual stimulus contrast for which participants were at chance at discriminating the prime category. This stimulus contrast was used in the main experiment. Two aspects of this procedure, however, suggest that partial awareness may have occurred. First, the awareness-check block was run before the experimental trials, so that participants were more practiced for trials in which priming was measured than for trials in which conscious perception was assessed. As perception of the prime is likely to increase with practice (e.g., Schwiedrzik et al., 2009), partial awareness of the prime cannot be excluded. In addition, the authors adopted a rather lenient criterion for consciousness: forced-choice discrimination performance ranging between 35 and 65% was held to reflect chance performance.

Faivre et al. (2012) reported unconscious priming elicited by CFS-suppressed faces. A significant improvement over previous study is that priming and conscious perception were measured under exactly the same conditions. The finding of Faivre et al.’s (2012) study is not necessarily incompatible with our results, however. While we found no priming for prime and target arrows that were physically different from each other, Faivre et al. (2012) reported priming in the form of a performance cost when the prime and target were identical, suggesting that sensory adaptation may have occurred. By contrast, they found that the same faces did not bias affective judgments of a subsequent neutral target. Taken together, these findings suggest that CFS suppression may allow very low-level perceptual processing of the prime but not response priming. Further research is required to further test this hypothesis.

Finally, Izatt et al. (2014) provided only weak evidence of unconscious priming by CFS-suppressed faces. First, their stimuli were considered to be invisible when subjects reported either no experience or a brief glimpse of the stimulus, that is, in conditions that are equivalent to visibility levels 0 and 1 of the present study. Considering that we found unconscious priming to be significant for visibility 1 (but not for null visibility), any unconscious priming demonstrated when these two visibility levels are collapsed may have resulted from partial awareness. Second, one could infer that unconscious priming occurred during CFS only from the fact that unconscious priming was significant across masking conditions (backward masking and CFS) and did not interact with masking technique. Thus, there was no direct test of unconscious priming by CFS-suppressed stimuli.

### METHODOLOGICAL IMPLICATIONS FOR THE STUDY OF UNCONSCIOUS PROCESSING

Our findings show that in the search for the boundaries of unconscious processing, the method used to prevent conscious perception matters: failure to observe that some process can be performed without conscious perception with one method does not necessarily entail that conscious perception is necessary for this process. In the present study, for instance, identification of the prime shape and activation of the motor response associated with this shape were found to be largely independent of conscious perception: priming was of the same magnitude when the prime was subjectively invisible, barely visible or almost clearly visible (although priming was further boosted when visibility was maximal). Yet, had one relied on the findings resulting from preventing conscious perception using CFS, the conclusion would have been that shape processing and/or motor preparation require conscious perception.

It follows that the best-suited methods to study unconscious processing are those that can entirely prevent conscious perception while minimally disrupting unconscious processing. In order to uncover such methods, different means of suppressing conscious vision must be compared. We suggest that such comparison is possible only if one ensures that the critical stimuli are equally close to the limen of consciousness for each of the compared methods. (Here, we defined conscious perception at the most basic level, namely, with regard to perception of the critical stimulus’ mere presence rather than with regard to perception of one of its features). We further suggest that a fruitful approach to measure distance from the limen is to use stimulus and temporal parameters that are associated with liminal perception and to assess conscious perception using a sensitive subjective scale. In this way, the distance from the limen can be estimated as the percentage of 0-visibility trials^6^. Again, it should be noted that objective measures of conscious perception cannot provide an estimate of such distance, as explained in the introduction. Here, we showed that although stimuli rendered invisible using CFS and meta-contrast masking (Experiment 1 vs. 3) were equally distant from the limen and produced similar priming effects for maximum visibility trials, unconscious response priming was large with one method and absent with the other.

## Conflict of Interest Statement

The authors declare that the research was conducted in the absence of any commercial or financial relationships that could be construed as a potential conflict of interest.

## Footnotes

1 Subjective measures of conscious perception have been criticized, based on the claim that they may be contaminated by responses biases – a problem that is often referred to as the criterion problem (e.g., Eriksen, 1960; Holender, 1986). However, recent research suggests that using a sensitive subjective measure may circumvent this problem. In a recent study (Peremen and Lamy, 2014) we used meta-contrast masking to manipulate conscious perception of a prime arrow pointing either to the left or to the right and measured conscious perception using a 0-to-3 subjective visibility scale. On trials in which the prime arrow was rated to be completely invisible (rating 0), objective performance at discriminating its direction fell to chance. By contrast, a rating of 1, indicating very faint visibility of the prime, was associated with above-chance performance. Focused scrutiny of the literature reveals that chance forced-choice performance at discriminating a simple feature of a target rendered invisible by a variety of methods, is not uncommon (e.g., Wyart and Tallon-Baudry, 2008; Bahrami et al., 2010). Taken together, these findings suggest that subjective reports of conscious perception can be as sensitive as measures relying on objective discrimination performance.

2 This experiment was reported in a previous paper (Peremen and Lamy, 2014, Experiment 2) to address a different question. Specifically, while in that paper the emphasis was on the comparison between subjective and objective measures of conscious perception, the emphasis here is on the comparison of unconscious processing during meta-contrast masking and continuous flash suppression.

3 The use of a go no-go task is related to the goals of Peremen and Lamy (2014) study and is irrelevant here. It will therefore not be considered further.

4 The distribution of visibility ratings could considerably vary between observers. However, we chose not to exclude subjects based on considerations of balanced visibility rating distribution. Instead, in order to overcome the distortions that might result from unbalanced repeated measures data, we used a mixed effects model to analyze the data when visibility was as a factor. Importantly, however, for this and the following experiments, the results remained the same when subjects with unbalanced distributions (fewer than 10% or more than 65% of 0-visibility trials) were excluded.

5 The relatively high mean RT in this and the next experiments (>600 ms) are likely to result from the dual-task situation. Consistent with this conjecture, we recently showed that when subjects had to respond to both the target and prime (as in the present experiments), RTs were on the order of 650 ms, whereas when they had to respond only to the target (all other things being identical), their RTs fell below 400 ms (Peremen and Lamy, 2014).

6 One could argue that the criterion used when reporting visibility of the prime may have been more conservative in the meta-contrast masking than in the CFS experiment. In other words, participants may have reported 0 visibility despite partial awareness of the prime in the former but not in the latter experiment. However, the findings of Peremen and Lamy (2014) study clearly argue against this claim. Using exactly the same stimuli and SOAs as in Experiment 1, we showed that participants were at chance at discriminating the direction of the prime arrow when reporting 0 visibility: had meta-contrast masking induced a conservative criterion, performance should have been above chance.
