# Supplementary material for: Comparing unconscious processing during continuous flash suppression and meta-contrast masking just under the limen of consciousness
Source: Front Psychol. 2014 Sep 11;5:969. doi: 10.3389/fpsyg.2014.00969 (PMC4160875; doi:10.3389/fpsyg.2014.00969)
Supplement: Supplementary file 1 [file Table_1.DOCX]

| **Visibility** | **SOA = 0ms** | | **SOA = 24ms** | | **SOA = 47ms** | | **SOA = 71ms** | | **SOA = 94ms** | | **SOA = 118ms** | |
| --- | --- | --- | --- | --- | --- | --- | --- | --- | --- | --- | --- | --- |
|  | *Congruent* | *Incongruent* | *Congruent* | *Incongruent* | *Congruent* | *Incongruent* | *Congruent* | *Incongruent* | *Congruent* | *Incongruent* | *Congruent* | *Incongruent* |
| **0** | 628.2 | 697.3 | 622.6 | 631.3 | 591.5 | 644.0 | 593.1 | 618.7 | 616.6 | 681.0 | 612.2 | 724.1 |
| **1** | 660.2 | 730.1 | 637.4 | 650.2 | 653.0 | 615.0 | 590.2 | 656.5 | 648.1 | 710.7 | 683.4 | 785.2 |
| **2** | 747.6 | 746.8 | 667.8 | 702.8 | 676.0 | 668.3 | 631.2 | 694.9 | 681.4 | 777.5 | 756.7 | 850.8 |
| **3** | 679.9 | 782.1 | 613.8 | 750.6 | 622.0 | 672.8 | 594.8 | 670.1 | 626.8 | 700.4 | 708.7 | 817.7 |

Supplementary Table 1: Mean reaction times (in milliseconds) on congruent and incongruent trials by SOA and visibility rating in Experiment 1.
