# Supplementary material for: Comparing unconscious processing during continuous flash suppression and meta-contrast masking just under the limen of consciousness
Source: Front Psychol. 2014 Sep 11;5:969. doi: 10.3389/fpsyg.2014.00969 (PMC4160875; doi:10.3389/fpsyg.2014.00969)
Supplement: Supplementary file 2 [file Table_2.DOCX]

Supplementary Table 2: Mean accuracy rates (in percentage) on congruent and incongruent trials by SOA and visibility rating in Experiment 1.

| **Visibility** | **SOA = 0ms** | | **SOA = 24ms** | | **SOA = 47ms** | | **SOA = 71ms** | | **SOA = 94ms** | | **SOA = 118ms** | |
| --- | --- | --- | --- | --- | --- | --- | --- | --- | --- | --- | --- | --- |
|  | *Congruent* | *Incongruent* | *Congruent* | *Incongruent* | *Congruent* | *Incongruent* | *Congruent* | *Incongruent* | *Congruent* | *Incongruent* | *Congruent* | *Incongruent* |
| **0** | 98.50% | 99.62% | 98.15% | 97.10% | 98.80% | 99.09% | 98.21% | 98.71% | 98.69% | 93.53% | 100.42% | 97.74% |
| **1** | 98.54% | 97.82% | 99.83% | 98.42% | 99.21% | 98.79% | 99.13% | 98.25% | 98.01% | 98.24% | 99.21% | 97.59% |
| **2** | 99.42% | 99.29% | 97.57% | 96.05% | 99.52% | 98.86% | 98.45% | 99.10% | 97.33% | 98.68% | 96.67% | 96.79% |
| **3** | 97.10% | 93.92% | 96.18% | 96.85% | 96.45% | 98.22% | 97.10% | 94.91% | 98.35% | 96.56% | 96.65% | 95.93% |
