# Supplementary material for: Comparing unconscious processing during continuous flash suppression and meta-contrast masking just under the limen of consciousness
Source: Front Psychol. 2014 Sep 11;5:969. doi: 10.3389/fpsyg.2014.00969 (PMC4160875; doi:10.3389/fpsyg.2014.00969)
Supplement: Supplementary file 3 [file Table_3.DOCX]

Supplementary Table 3: Mean reaction times (in milliseconds) on congruent and incongruent trials by SOA and visibility rating in Experiment 2.

| **Visibility** | **SOA = 250ms** | | **SOA = 350ms** | | **SOA = 450ms** | | **SOA = 550ms** | | **SOA = 650ms** | |
| --- | --- | --- | --- | --- | --- | --- | --- | --- | --- | --- |
|  | *Congruent* | *Incongruent* | *Congruent* | *Incongruent* | *Congruent* | *Incongruent* | *Congruent* | *Incongruent* | *Congruent* | *Incongruent* |
| **0** | 713.8 | 710.1 | 707.2 | 697.6 | 664.0 | 665.2 | 665.5 | 682.7 | 657.2 | 676.3 |
| **1** | 787.4 | 803.8 | 769.0 | 785.1 | 718.3 | 771.6 | 752.9 | 768.0 | 739.1 | 760.3 |
| **2** | 783.0 | 832.1 | 781.3 | 820.7 | 740.3 | 801.2 | 730.0 | 750.9 | 735.9 | 770.0 |
| **3** | 761.5 | 800.7 | 723.3 | 752.0 | 696.2 | 764.5 | 694.2 | 743.1 | 669.7 | 715.7 |
