# Supplementary material for: Comparing unconscious processing during continuous flash suppression and meta-contrast masking just under the limen of consciousness
Source: Front Psychol. 2014 Sep 11;5:969. doi: 10.3389/fpsyg.2014.00969 (PMC4160875; doi:10.3389/fpsyg.2014.00969)
Supplement: Supplementary file 4 [file Table_4.DOCX]

Supplementary Table 4: Mean accuracy rates (in percentage) on congruent and incongruent trials by SOA and visibility rating in Experiment 2.

| **Visibility** | **SOA = 250ms** | | **SOA = 350ms** | | **SOA = 450ms** | | **SOA = 550ms** | | **SOA = 650ms** | |
| --- | --- | --- | --- | --- | --- | --- | --- | --- | --- | --- |
|  | *Congruent* | *Incongruent* | *Congruent* | *Incongruent* | *Congruent* | *Incongruent* | *Congruent* | *Incongruent* | *Congruent* | *Incongruent* |
| **0** | 98.99% | 98.84% | 99.99% | 99.37% | 99.49% | 99.99% | 99.48% | 98.72% | 99.72% | 97.81% |
| **1** | 98.58% | 99.20% | 99.24% | 96.20% | 99.04% | 97.05% | 99.03% | 99.02% | 98.82% | 97.26% |
| **2** | 99.98% | 99.97% | 98.75% | 95.41% | 100.03% | 95.73% | 99.98% | 96.93% | 98.94% | 94.53% |
| **3** | 99.26% | 99.29% | 98.88% | 99.43% | 99.17% | 98.12% | 98.82% | 97.44% | 99.66% | 96.28% |
