# Supplementary material for: Comparing unconscious processing during continuous flash suppression and meta-contrast masking just under the limen of consciousness
Source: Front Psychol. 2014 Sep 11;5:969. doi: 10.3389/fpsyg.2014.00969 (PMC4160875; doi:10.3389/fpsyg.2014.00969)
Supplement: Supplementary file 5 [file Table_5.DOCX]

Supplementary Table 5: Mean reaction times (in milliseconds) on congruent and incongruent trials by contrast level and visibility rating in Experiment 2.

| **Visibility** | **Contrast = 20%** | | **Contrast = 60%** | | **Contrast = 100%** | |
| --- | --- | --- | --- | --- | --- | --- |
|  | *Congruent* | *Incongruent* | *Congruent* | *Incongruent* | *Congruent* | *Incongruent* |
| **0** | 684.3 | 686.5 | 687.5 | 686.2 | 685.1 | 695.5 |
| **1** | 751.9 | 762.1 | 769.6 | 782.0 | 751.3 | 793.8 |
| **2** | 744.6 | 792.5 | 753.2 | 787.6 | 753.4 | 793.6 |
| **3** | 703.8 | 733.5 | 706.6 | 763.0 | 684.5 | 740.2 |
