# Supplementary material for: Comparing unconscious processing during continuous flash suppression and meta-contrast masking just under the limen of consciousness
Source: Front Psychol. 2014 Sep 11;5:969. doi: 10.3389/fpsyg.2014.00969 (PMC4160875; doi:10.3389/fpsyg.2014.00969)
Supplement: Supplementary file 6 [file Table_6.DOCX]

Supplementary Table 6: Mean accuracy rates (in percentage) on congruent and incongruent trials by contrast level and visibility rating in Experiment 2

| **Visibility** | **Contrast = 20%** | | **Contrast = 60%** | | **Contrast = 100%** | |
| --- | --- | --- | --- | --- | --- | --- |
|  | *Congruent* | *Incongruent* | *Congruent* | *Incongruent* | *Congruent* | *Incongruent* |
| **0** | 99.74% | 99.34% | 99.40% | 98.70% | 99.37% | 98.90% |
| **1** | 97.79% | 98.48% | 98.90% | 97.11% | 100.03% | 97.57% |
| **2** | 98.63% | 97.09% | 100.00% | 96.58% | 99.95% | 95.48% |
| **3** | 99.30% | 98.08% | 99.50% | 98.10% | 98.78% | 97.41% |
